# The importance of the thickness of femoral lateral wall for treating intertrochanteric fractures: a finite elements analysis

**DOI:** 10.1038/s41598-023-39879-9

**Published:** 2023-08-04

**Authors:** Shuang Li, Zhi-hao Su, Jia-min Zhu, Wan-ju Sun, Yi-Chen Zhu, Jian Wang, Kai Li, Ming Ni, Shuai Han

**Affiliations:** 1 Department of Orthopaedic Surgery, Pudong New Area Peoples’ Hospital, No. 490 Chuanhuan South Road, Pudong New Area, Shanghai, 201299 People’s Republic of China; 2 School of Health Science and Engineering, University of Shanghai for Science and Technology, Shanghai, 200093 China; 3 Department of Orthopaedic Surgery, Ruijin Hospital, Shanghai Jiao Tong University School of Medicine, Shanghai, 200025 China

**Keywords:** Anatomy, Medical research, Experimental models of disease

## Abstract

To explore how the thickness of the femoral lateral wall influences the effectiveness of internal fixation systems used to treat intertrochanteric fractures. CT images of the pelvis and femur of a male adult were used to construct an intertrochanteric fracture model (AO/OTA 31-A2) with various thicknesses of the femoral lateral wall (FLW). Four finite element (FE) models were created with the lateral femoral walls being 10 mm, 20 mm, 30 mm, and 40 mm thick. The fracture models were fixed with a dynamic hip screw (DHS), a proximal femoral nail anti-rotation (PFNA), and a proximal femoral locking compression plate (P-FLCP). A simulated vertical load was applied to the femoral head. The stress and displacement of the implant and femur in each model were recorded for comparison. The FE analysis of the intertrochanteric fracture models showed that the PFNA system could provide better stability than the DHS and P-FLCP with the same thickness of FLW. The FLW provided buttress support to the femoral head and neck when using a DHS and PFNA, and the buttress strength was proportional to the thickness of FLW. The maximum stress in the DHS model was recorded on the DHS plate which accommodated the lag screw. For the PFNA model, the maximum stress appeared at the connection between the nail and blade. In the P-FLCP model, the maximum stresses were highly concentrated at the connection between the cephalic nails and the proximal plate. The thickness of the femoral lateral wall should be considered an important factor when selecting a suitable internal fixation system for intertrochanteric fractures. Based on the FE analysis, intramedullary fixation, such as PFNA, experiences lower stress levels and a moderate displacement in comparison to DHS and P-FCLP when used to treat intertrochanteric fractures.

## Introduction

Intertrochanteric fractures (ITF) are commonly seen in clinical practice, particularly in the elderly. For most ITF patients, surgical intervention is the standard treatment for pain relief and to regain joint movement^1^. Dynamic hip screws (DHSs) are one of the most common fixation devices for AO/OTA 31-A1 and partial A2 fractures with an intact lateral femoral wall. For unstable fractures, fixation with intramedullary nails such as the proximal femoral nail anti-rotation (PFNA) has been shown to be a reliable and effective method for treating an ITF^2^. However, when there is an associated lateral wall fracture, intramedullary nails have been associated with higher failure and revision rates regardless whether extra- or intra-fixation is used^3^.

The composition and thickness of the femoral lateral wall (FLW) has been suggested to be a major risk factor for ITF. Through retrospective analysis, Hsu et al.^4^ determined a lateral wall thickness of 20.5 mm to be threshold value for postoperative lateral wall fracture and Palm et al.^5^ reported that the integrity of the lateral femoral wall is a predictor of the need for reoperation. To the author's knowledge, there is a paucity of literature regarding the thickness of the FLW and subsequent reduction with different fixation systems.

Therefore, the objective of this study was to assess different thicknesses of the FLW in ITF secured with an intra-medullary nail and extra-medullary fixation. To our knowledge, this is the first study to investigate the stability of different implants for treating intertrochanteric fractures while considering the thickness of the lateral wall.

## Materials and methods

### Ethical approval

The study was approved by the institutional ethics committee of Pudong New Area Peoples’ Hospital (approval number: 2021K29). All experiments were performed in accordance with the Declaration of Helsinki. Informed consent was obtained and signed by the subject prior to participation in this study.

### General study design

We hypothesized that anatomical reduction and a robust femoral lateral wall (FLW) are important to reduce the risk of complications following implantation of a buttress plate or PFNA. A thick FLW can increase the stiffness of the bone-implant construct.

### Finite element modeling

A CT scanner (Philips, Brilliance 64) was used to capture 64 images of the pelvis and femur from a 45 years old healthy male adult. The slice thickness of the CT images was 1.25 mm, with a resolution of 512 × 512 pixels. The DICOM images were imported into Mimics 18.0 software (Materialise NV Technologielaan, Leuven, Belgium) to outline the inner and outer contours of the cortical bone. A threshold of 600 Hounsfield units was used to define the boundaries of the cortical shell and cancellous core^6^. CAD models of a DHS, PFNA, and proximal femoral locking compression plate (P-FLCP) were created in Solidworks 2014 (Dassault Systèmes, Vélizy-Villacoublay, France) according to published specifications from the WEGO ORTHO Corporation^7^. The implants were incorporated into the femoral model (AO/OTA 31-A2.3 fracture) using Abaqus 6.13 (Dassault Systèmes, Vélizy-Villacoublay, France).

Then the finite element (FE) models were created. The Young’s modulus was set at 17,000 MPa for cortical bone and 260 MPa for cancellous bone^8^. All implants were assigned material properties of TiAl6V4, with a Young’s modulus of 110,000 MPa and Poisson’s ratio of 0.33^8^. All materials were assumed to be homogeneous, isotropic and linearly elastic. The friction coefficient was 0.46 for bone–bone interactions, 0.42 for bone–implant interactions and 0.2 for implant–implant interactions^9^. All implanted models used four tetrahedron type elements, with about 45,000 elements and 100,000 nodes in each model (Table 1).

**Table 1:** Quantity of elements and nodes in the three implant models.

Thickness of FLW (mm)	Element			Node		
	DHS	P-FLCP	PFNA	DHS	P-FLCP	PFNA
10	470,649	437,820	438,215	104,211	98,075	96,504
20	467,139	438,667	442,203	103,581	98,317	97,416
30	455,010	436,907	441,970	101,087	97,904	97,338
40	434,208	435,315	440,242	96,891	97,510	96,866

**Figure 1:**
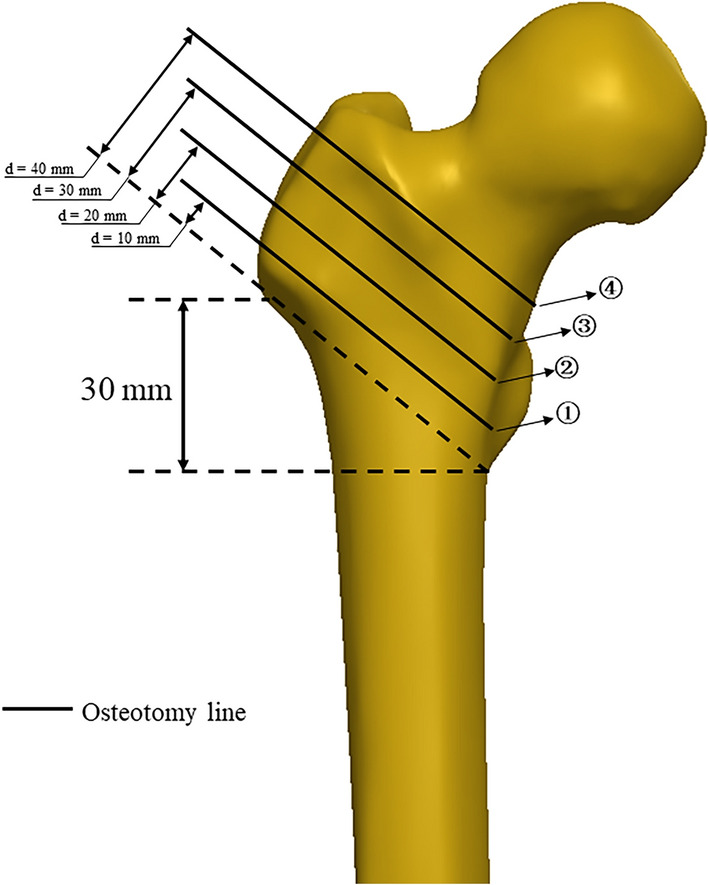
Schematic diagram of the osteotomy lines used to produce the intertrochanteric fracture models with different thicknesses of the lateral femoral wall. The lateral wall thickness (d) is defined as the distance in millimeters (mm) from a reference point 3 cm below the innominate tubercle of the greater trochanter to a line angled at 135° to the fracture line on the anteroposterior X-ray. The first osteotomy line was created by linking the apex of the greater trochanter and the base of the lesser trochanter on the frontal plane, which corresponded to the first fracture model (①) with a measured lateral femoral wall thickness of 10 mm. The second osteotomy line was created by rotating the first osteotomy line around the base of the lesser trochanter to produce a lateral femoral wall thickness of 20 mm for the second fracture model (②). The other osteotomy lines and fracture models (③ and ④) were constructed by repeating the second step and rotating the line in 10 mm intervals.

**Figure 2:**
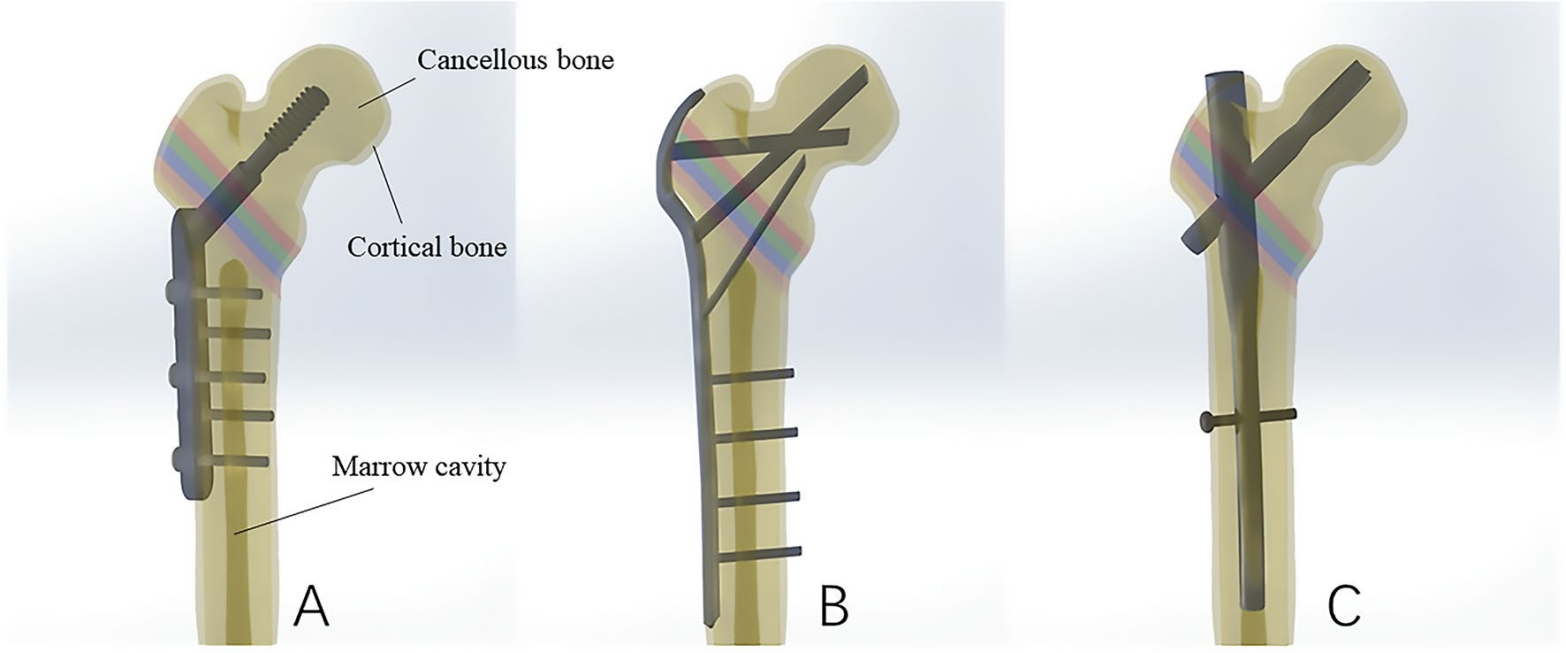
Schematic diagram of the assembly of three bone-implant construct models. (**A**) DHS; (**B**) P-FLCP; (**C**) PFNA.

As shown in Fig. 1, the lateral wall of the femur was defined as the span from the lateral cortex of the proximal femur to the vastus ridge. The lateral wall thickness was defined as the distance from a reference point 30 mm below the innominate tubercle of the greater trochanter to the fracture line angled at 135° on the antero-posterior X-ray. The first osteotomy line was created by linking the apex of the greater trochanter and the base of the lesser trochanter on the frontal plane. Lines plotted parallel to the dotted line in Fig. 1 were used to assign the thickness of the FLW as 10 mm, 20 mm, 30 mm and 40 mm. The three bone-implant construct models were then assembled into the femurs with four different thicknesses of the FLW (Fig. 2).

### Mesh convergence test

A mesh convergence test was conducted on the DHS-bone construct model with the FLW set at 10 mm, 20 mm, 30 mm, and 40 mm. The test used the same boundary conditions and was performed under a compression load of 1800 N. The test was conducted by reducing the mesh size by about 20% (global mesh size from 2.5 to 2.0 mm). Model convergence was achieved with the final mesh size of 2.0 mm, where the von Misses stress varied by less than 2% with further reductions in the cell size^10^.

### Boundary and loading conditions

**Figure 3:**
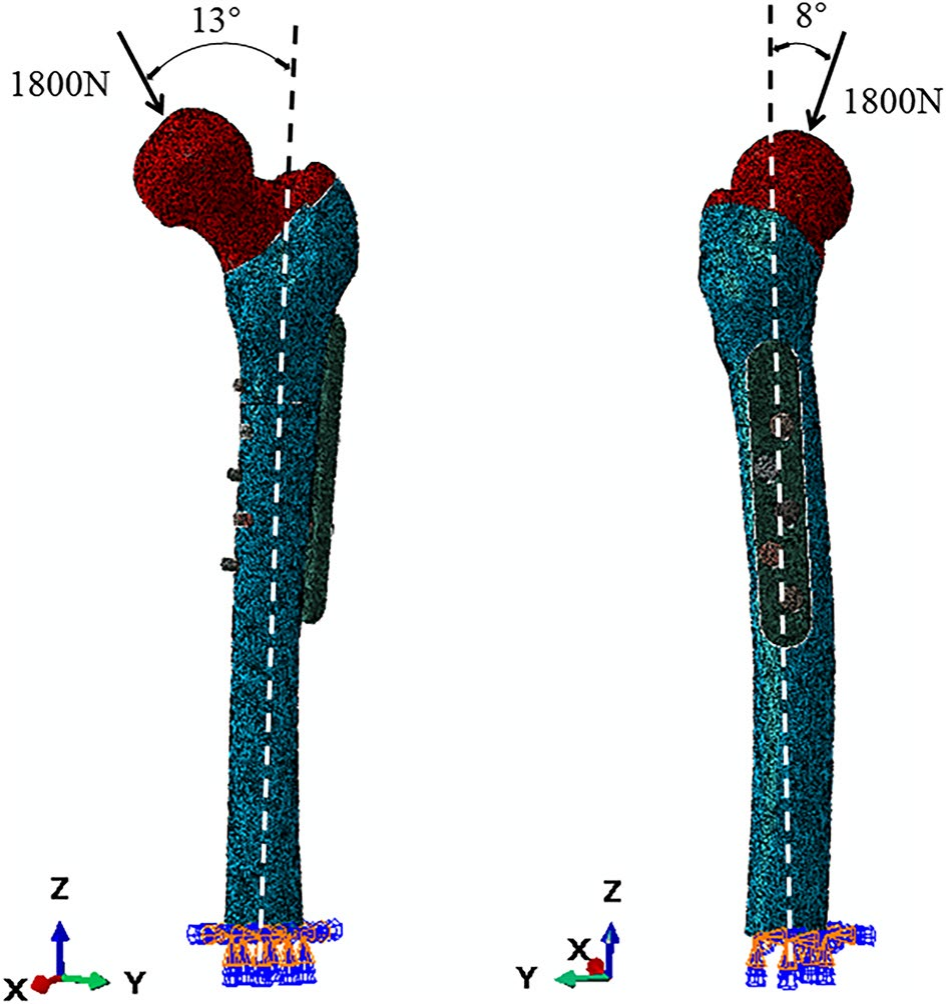
Diagram of the boundary conditions of the FE model showing the vector of a single-stance hip joint force (F) with angles of action in the frontal plane (A) and the sagittal plane (B).

The implanted femoral FEA model was fully fixed (zero displacement) at the distal end. Using Abaqus 6.13, the femur was subjected to a compression load of 1800 N applied at an angle of 13° in adduction in the frontal plane and 8° in the sagittal plane to simulate anatomical loading during single-leg stance^11,12^ (Fig. 3). The maximum von stress and displacement of the models were recorded^13^.

### FE model validation

**Figure 4:**
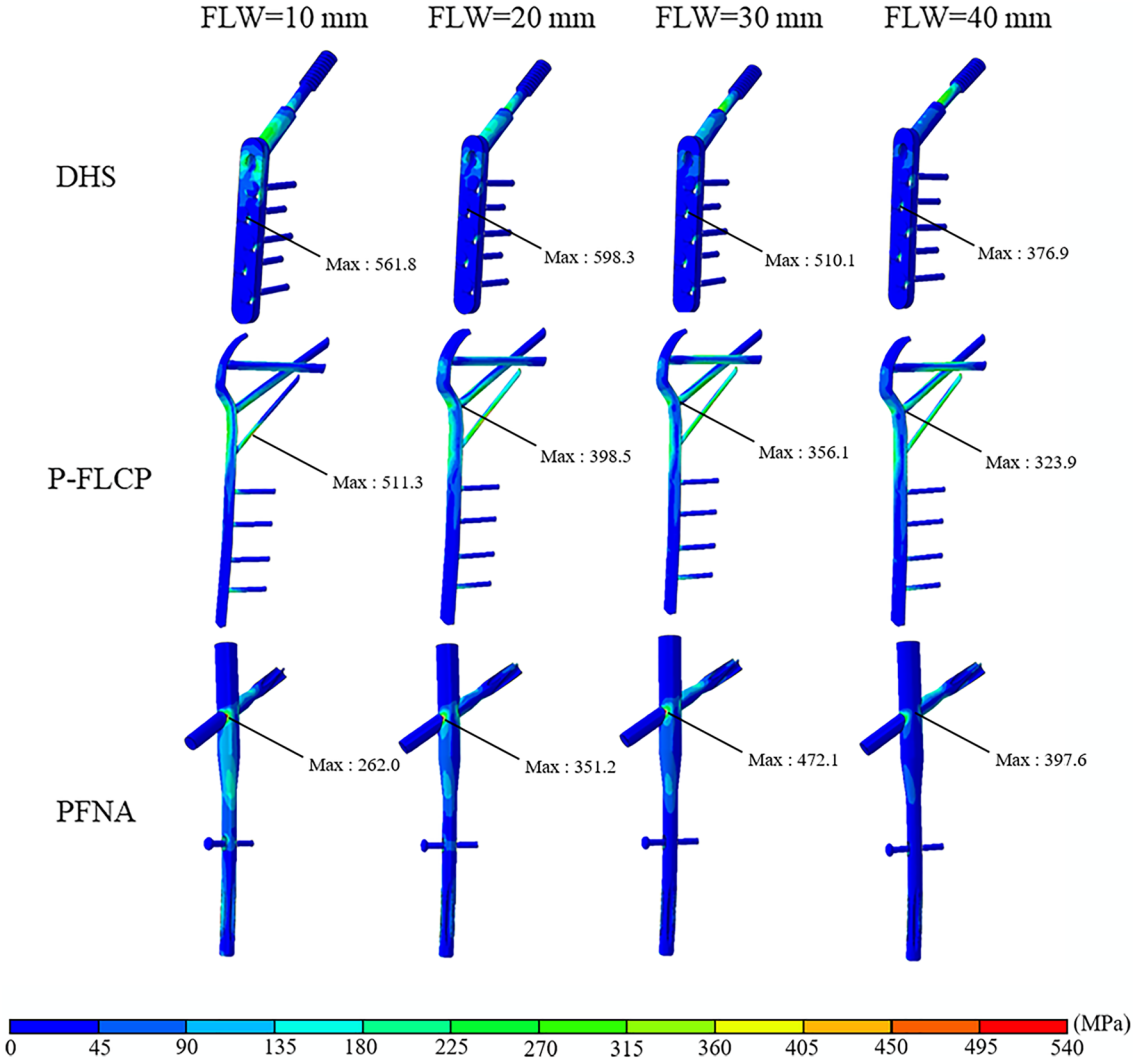
Von Misses stress (MPa) on the bone-implant construct when placed under a load of 1800 N. The first row is DHS, the middle row is P-FLCP and the last row is PFNA. The first column shows models with FLW = 10 mm, 2nd column with FLW = 20 mm, 3rd column with FLW = 30 mm, and 4th column with FLW = 40 mm.

**Figure 5:**
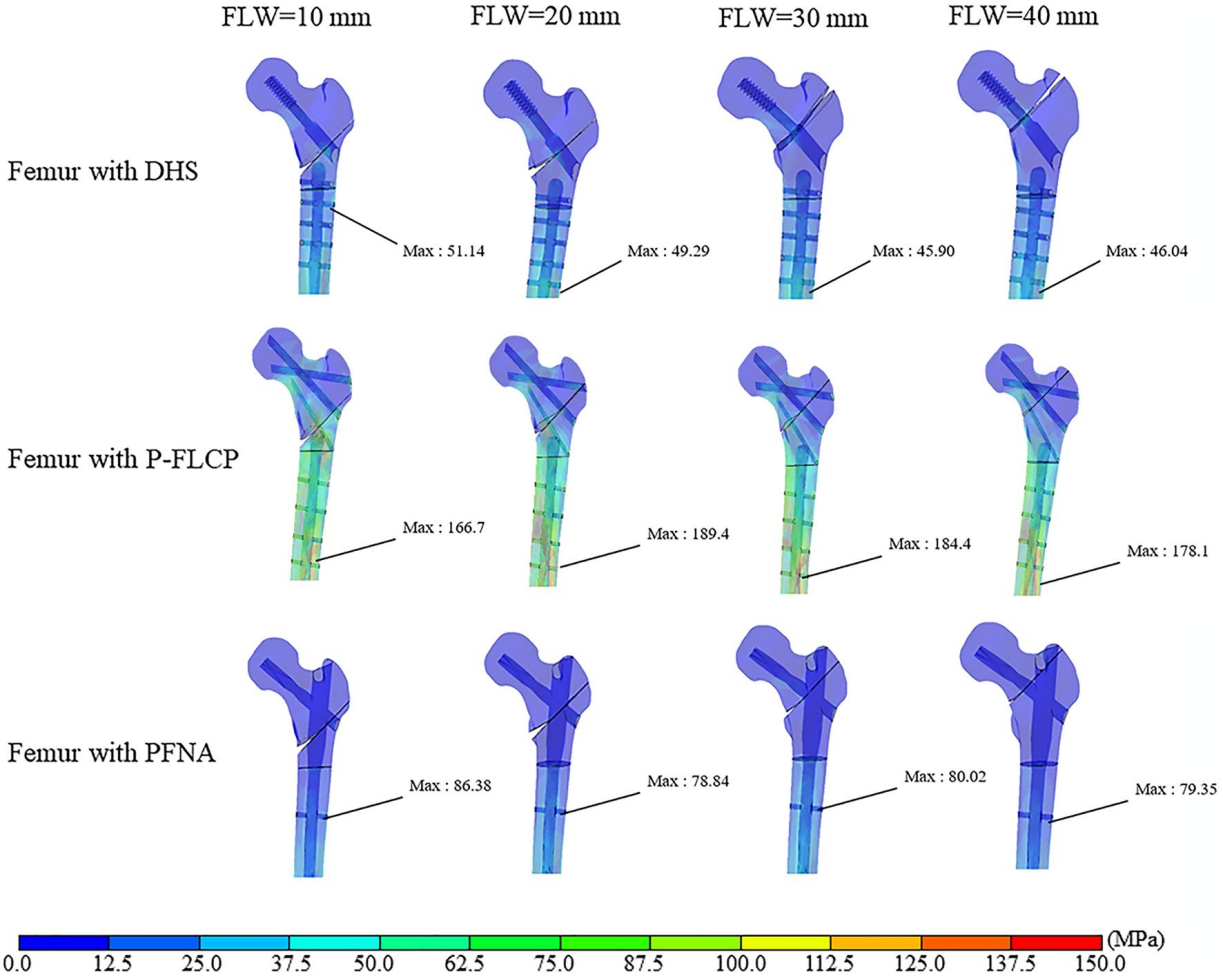
Von Misses stress (MPa) on the synthetic femoral bone when placed under a load of 1800 N. The first row is the DHS group, the middle row is the P-FLCP group, and the last row is the PFNA group. The first column shows models with FLW = 10 mm, 2nd column with FLW = 20 mm, 3rd column with FLW = 30 mm, and 4th column with FLW = 40 mm.

**Figure 6:**
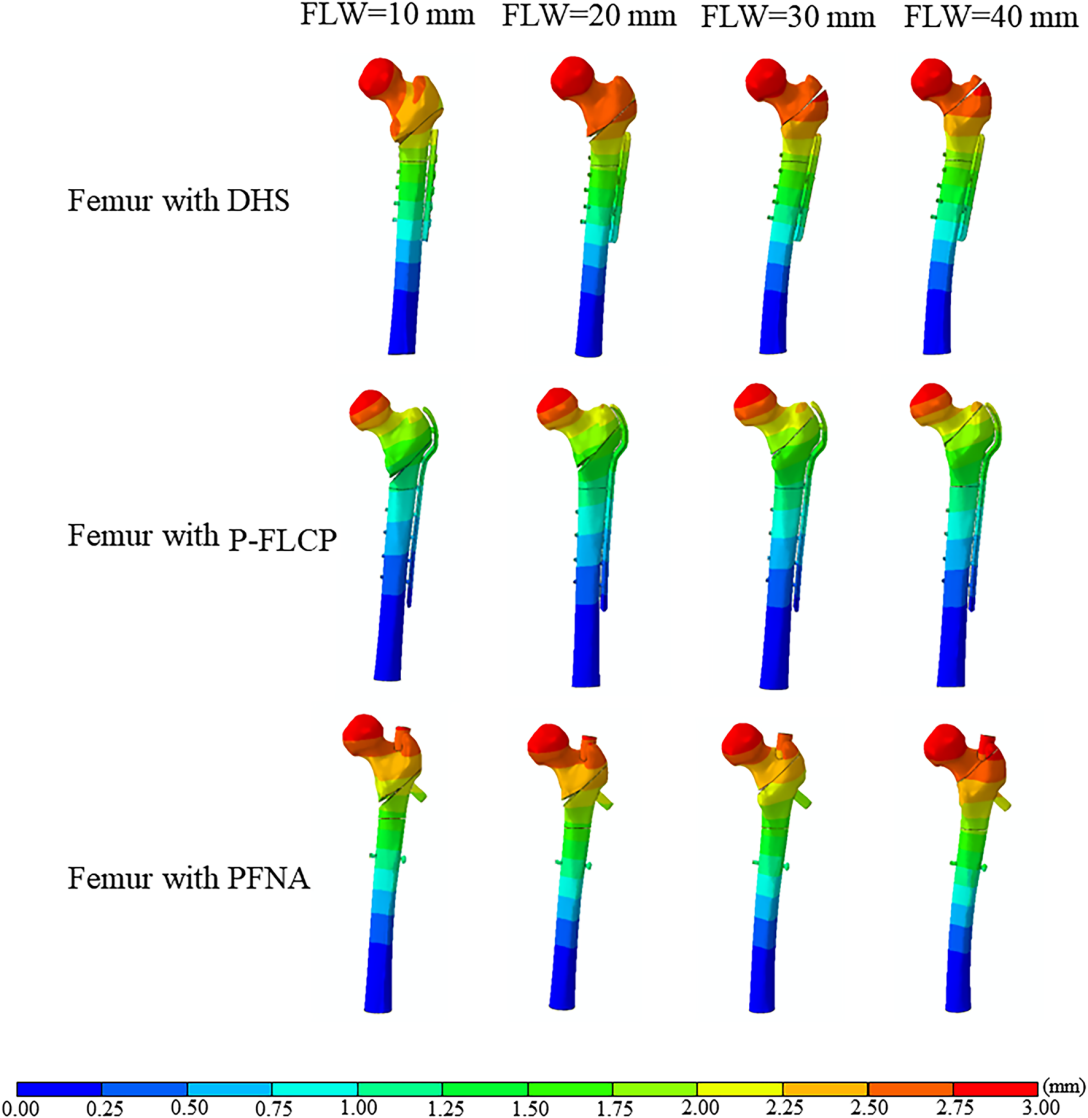
The maximum displacement of the fracture position for the three bone-implant construct models. The first row is the DHS group, the middle row is the P-FLCP group, and the last row is the PFNA group. The first column shows models with FLW = 10 mm, 2nd column with FLW = 20 mm, 3rd column with FLW = 30 mm, and 4th column with FLW = 40 mm.

**Figure 7:**
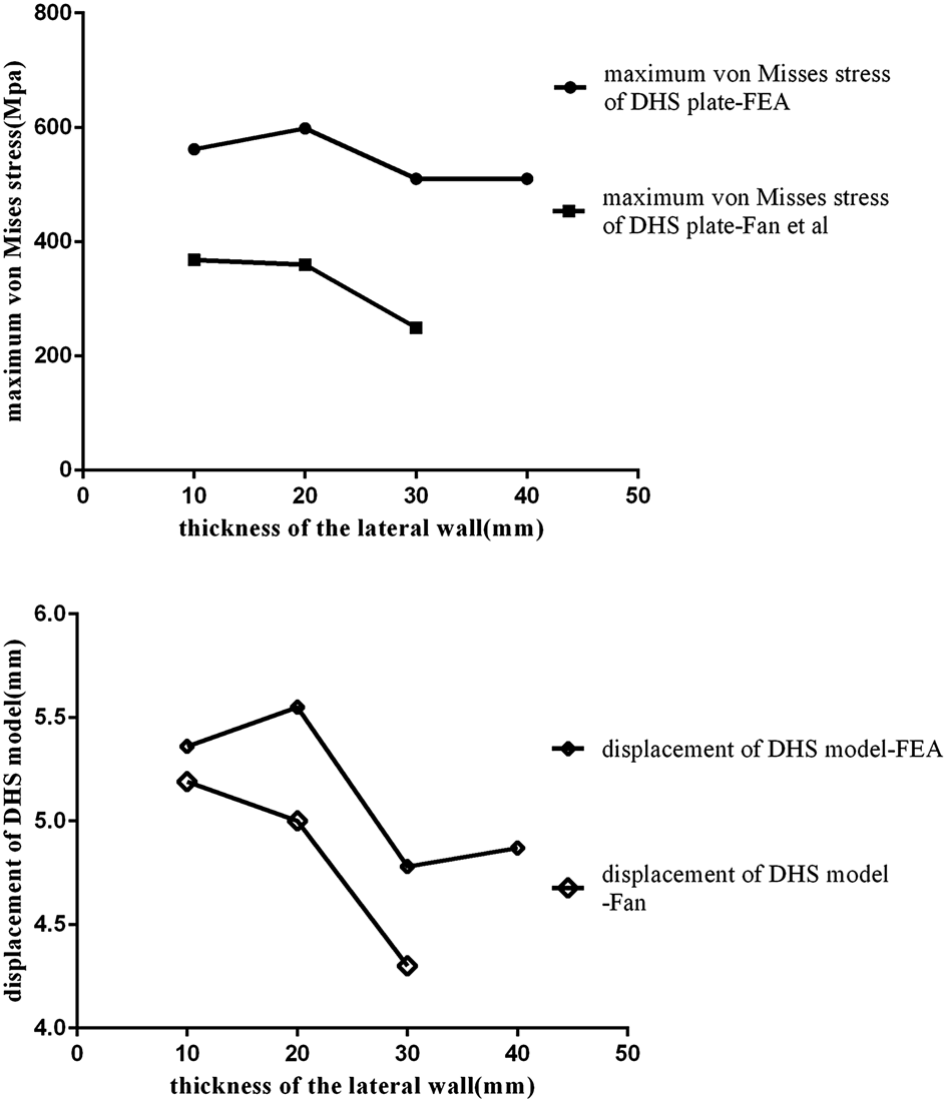
Comparison of stress and displacement between the current FEA study and a previous study. Similar variations in stress and displacement confirm the validity of the FEA models^16^.

The FE model in this current study was validated in previous studies and shown to reliably predict the biomechanical performance of DHS, PFNA and P-FLCP implants^7,14,15^. Figures 4 and 5 compare the stress and displacement between the implants simulated in this current study. Figures 6 and 7 show that the variations in stress and displacement with the DHS, PFNA and P-FLCP implants were similar in the previous studies^7,15,16^.

### Statistics

Given the descriptive study design, no statistical tests were applied. However, the results show a clear comparison between the stress and displacement at the femoral fragment and implants.

## Results

### Von Mises stress distribution on the implants

Figure 4 shows the von Mises stress distribution on the implants. For DHS fixation, the maximum stresses were mostly concentrated at the lateral side of the proximal screw hole of the DHS and the lowest von Mises stress was at the tip of the distal screw. For P-FLCP fixation, the maximum stresses were located at the connection between the cephalic nails and the proximal plate, and the lowest stresses were found from the tip of the fourth screw to the distal end of the implant. For PFNA fixation, the maximum stress appeared at the connection between the nail and the blade. With a 10 mm FLW, the lowest stress appeared at the tip of the distal screw, but shifted to the proximal cephalic nail when the FLW was increased to 20 mm, 30 mm, and 40 mm. As shown in Table 2, the maximum von Mises stress on DHS and P-FLCP implant decreased as the FLW became thicker (10–40 mm), ranging from 561.8 to 376.9 MPa for the DHS plate, and from 511.3 to 323.9 MPa for the P-FLCP plate. Because of unique PFNA structure (short arm of force and large contact surface), the maximum von Mises stress on PFNA implant was a more moderate distribution (262.0 MPa to 397.6 MPa) as the FLW thickness increases.

**Table 2:** The maximum von Misses stress and total displacement of DHS, P-FLCP and PFNA model.

Thickness of FLW (mm)	Maximum von Misses stress (MPa)			Total displacement (mm)		
	DHS plate	P-FLCP	PFNA	DHS	P-FLCP	PFNA
10	561.8	511.3	262.0	5.36	10.88	6.80
20	598.3	398.5	351.2	5.55	9.57	6.53
30	510.1	356.1	472.1	4.78	9.13	6.08
40	376.9	323.9	397.6	4.87	8.78	5.37

### Von Mises stress distribution on synthetic femoral bone

Figure 5 depicts the von Mises stress distribution on the synthetic femoral bone with simulated osteotomies secured using a DHS, P-FLCP, and PFNA devices. For the DHS fixation group, with a 10 mm FLW, the maximal stress was concentrated on the lateral side of the proximal part of the shaft but shifted towards the distal end with the 20 mm, 30 mm, and 40 mm FLW. The P-FLCP fixation group suffered greater stress than both the DHS and PFNA fixation groups, for which the stress values were similar.

### Displacement of the femoral-implant construct

The maximum displacements of the DHS, P-FLCP and PFNA implants and bones are shown in Fig. 6. Under the 1800 N compressive load, the total displacement of the DHS construct with a 10 mm FLW (5.36 mm) was 10.1% greater than with a 40 mm FLW (4.87 mm). Similarly, increasing the thickness of the FLW in the P-FLCP model from 10 to 40 mm resulted in a 19% reduction in implant total displacement, dropping from 10.88 to 8.78 mm. For the PFNA model, the implant total displacement reduced by 21% (from 6.80 to 5.37 mm) as the thickness of FLW was increased from 10 to 40 mm.

## Discussion

In this study, these FE models of a femur with simulated intertrochanteric fractures (ITF) were constructed to investigate how the thickness of the femoral lateral wall (FLW) influences the stability of the fracture after being treated with different fixation methods. The results showed that a femur with a thinner FLW has a greater risk of implant failure regardless of the fixation method used. Increasing the thickness of the FLW from 10 to 40 mm reduced the displacement of the DHS construct by about 10%, the P-FLCP by approximately 19%, and the PFNA by about 21%. As expected with the smaller change in displacement, the PFNA had the lowest stress values of the three implants and the stress was more evenly distributed across the implant. The DHS group had the greatest stress. The results of this study show that for a 31-A2 fracture placed under a 1800 N load, the displacement of the femoral bone was considerably lower with the PFNA implant than with P-FLCP, and the stress distribution of the femoral-implant construct was considerably lower with the PFNA implant than with DHS and P-FLCP.

This study found that the thickness of the FLW can be used to predict the integrity and stability of a reduced intertrochanteric fracture. The fracture was defined by a line starting from a point 3 cm inferior to the innominate tubercle of the greater trochanter and angled at 135° towards the coronal plane^17^, as illustrated in Fig. 1. The Orthopaedic Trauma Association Committee for Coding and Classification (AO/OTA) ranks fractures as being more unstable when the lateral femoral wall is less than 20.5 mm thick, which is justified by the rate of secondary fractures and complications^18^. Zheng et al.^19^ suggested that having a lateral wall thickness of less than 21.4 mm increases the risk of implant failure, while Hsu et al.^17^ found that a thickness of less than 20.5 mm may lead to premature failure and also that the fracture should not be fixed with a DHS alone. Li et al.^20^ advocated that having a residual lateral wall width of less than 18.55 mm is a reliable predictor of postoperative mechanical complications. As shown in Table 2, the FE analysis in this study showed that a lateral wall thickness of 20 mm was the threshold value for maintaining relatively low peak von Misses stress when using a PFNA.

From a clinical perspective, if the lateral wall is intact (AO/OTA subtype-31A1 and A2.1), the incidence of lateral wall fracture after surgery is low^21^. In such cases, a DHS implant is considered effective and safe for these types of fractures. When the lateral wall is vulnerable (AO/OTA subtype-31-A2.2 and A2.3), rupture of the iatrogenic lateral wall can occur during surgery, especially in osteoporotic patients. In these cases, the preference is to use a cephalomedullary nail or P-FLCP^22,23^. If the lateral wall is already fractured pre-operatively (AO/OTA subtype-31A3), the general consensus from surgeons is to use an intramedullary nail^24^.

The integrity of the lateral femoral wall is increasingly being recognized as an important consideration when treating intertrochanteric fractures. Few studies have investigated the importance of the lateral wall in ITF and how the wall thickness can influence fracture stability^25–27^. Joshi et al.^28^ found that 1.7% patients with a stable AO/OTA A1 fracture had iatrogenic lateral wall fractures, while 50% of patients with type A2.2 and type A2.3 also sustained an iatrogenic lateral wall fracture. They proposed that using a DHS with an unstable intertrochanteric fracture may increase the risk of an iatrogenic lateral wall fracture. However, Kim et al. reported that when using an intramedullary nail on A3.3 intertrochanteric fractures, the displaced lateral wall fragments tend to reduce spontaneously without any additional fixation during the postoperative period. They concluded that no additional fixation is needed for displaced lateral wall fragments after surgery with an intramedullary nail^29^.

Although previous studies focused on preoperative predictors of lateral wall fracture when using a DHS, none have done so for fixation with cephalomedullary nails such as the PFNA^14^. This study found stress concentrations at the bone-implant interface with the PFNA, which is consistent with literature^13^. The stress levels on the PFNA components were consistently higher than on the femoral bone in all simulated conditions. As the thickness of the lateral wall decreased, the stress on the PFNA construct transferred distally from the spiral blade to the locking screw. When the thickness of the lateral wall was decreased, the bone formed an interlocking structure with the PFNA nail that reduced the stress on the exterior-superior proximal femur, the proximal fragment of the femur, and the spiral blade. Furthermore, cephalomedullary nails are believed to be more appropriate for pertro-chanteric fractures that are accompanied by lateral wall fractures because the proximal end of the nails may act as the lateral wall to buttress the proximal fragments^18^.

Dai et al.^30^ reported a significantly lower mean pertrochanteric fracture height in patients with intra-operative lateral wall fractures than those without (15.6 mm and 28.5 mm). Dai reported that a threshold height of 20.445 mm is a reliable predictor of iatrogenic lateral wall fractures when using cephalomedullary nails. This should be considered by surgeons when choosing a suitable fixation method. Hsu et al.^16^ considered that the relatively low threshold may be caused by the thinner lateral wall and comminution of the postero-medial fragment in the presence of unstable fractures. Their results showed that the incidence of iatrogenic fractures was also significantly higher in A2 than A1 fractures (46.7% vs. 15.0%, respectively) when using cephalomedullary nails.

This study has some limitations. The data was taken from computational models created from images of a single male patient without considering influential biomechanical and biological factors of the typical femoral anatomy. Also, the measurements reported may not be entirely representative of other patient cohorts, for example females and the elderly. Future studies may consider additional factors, such as soft tissues and cartilage, pits, screw type, fracture type, and quality of bone. Another limitation is that the finite element models were meshed using a mesh size widely adopted in literature. Biomechanical testing should be used to determine the power for the specific models and to determine the level of discretization error.

## Conclusion

The results of this study show that the thickness of the femoral lateral wall should be assessed prior to surgery and be considered when selecting a suitable fixation implant for intertrochanteric fractures. Based on the FE analysis, intramedullary fixation, such as PFNA, demonstrates a lower stress level and moderate displacement compared to DHS and P-FCLP in the treatment of intertrochanteric fractures, taking into consideration the thickness of the femoral lateral wall.

## Data Availability

All the data will be available upon motivated request to the corresponding author of the present paper.

## Abbreviations

CT: Computed tomography
OTA/AO: Orthopaedic Trauma Association/Arbeitsgemeinschaft für Osteosynthesefragen
FLW: The femoral lateral wall
FEA: The finite element analysis
DHS: Dynamic hip screw
PFNA: Proximal femoral nail anti-rotation
P-FLCP: Proximal femoral locking compressing plate
